# Improved Classification of Blood-Brain-Barrier Drugs Using Deep Learning

**DOI:** 10.1038/s41598-019-44773-4

**Published:** 2019-06-19

**Authors:** Rui Miao, Liang-Yong Xia, Hao-Heng Chen, Hai-Hui Huang, Yong Liang

**Affiliations:** 1 Faculty of Information Technology, Macau University of Science and Technology, Avenida Wai Long, Taipa Macau, China; 2 School of Information Science and Engineering, Shaoguan University, No. 288, University Road, Zhenjiang District, Shaoguan City, Guangdong Province China; 3 State Key Laboratory of Quality Research in Chinese Medicines, Macau University of Science and Technology, Avenida Wai Long, Taipa Macau, China

**Keywords:** Computational models, Data mining, Machine learning, Predictive medicine

## Abstract

Blood-Brain-Barrier (BBB) is a strict permeability barrier for maintaining the Central Nervous System (CNS) homeostasis. One of the most important conditions to judge a CNS drug is to figure out whether it has BBB permeability or not. In the past 20 years, the existing prediction approaches are usually based on the data of the physical characteristics and chemical structure of drugs. However, these methods are usually only applicable to small molecule compounds based on passive diffusion through BBB. To deal this problem, one of the most famous methods is multi-core SVM method, which is based on clinical phenotypes about Drug Side Effects and Drug Indications to predict drug penetration of BBB. This paper proposed a Deep Learning method to predict the Blood-Brain-Barrier permeability based on the clinical phenotypes data. The validation result on three datasets proved that Deep Learning method achieves better performance than the other existing methods. The average accuracy of our method reaches 0.97, AUC reaches 0.98, and the F1 score is 0.92. The results proved that Deep Learning methods can significantly improve the prediction accuracy of drug BBB permeability and it can help researchers to reduce clinical trials and find new CNS drugs.

## Introduction

Currently, neurological diseases account for 28% of people with disabilities of all ages^1^. Despite the high prevalence associated with Central Nervous System (CNS) disease, effective medicines for these diseases are in scarcity. Researchers have done a lot of works on drug discovery. However, many tested compounds had failed due to lack of the ability to penetrate Blood–Brain-Barrier (BBB) rather than lack of potency, which made BBB get stuck in CNS drug discovery^2–7^. BBB is a special selective border with semi-permeability. This border can prevent certain substances (mostly harmful) from entering the brain tissues. BBB limit the passage of most of the external compounds (98%) to maintain CNS at the steady state^8^. Therefore, to determine a drug whether has BBB permeability is a pre-requirement of discovering CNS drugs^9–12^. Although the clinical experiment is the most accurate method of measuring BBB permeability^13^, it is difficult to do so due to the limitation of various types of drugs. Therefore, BBB permeability needs to be forecasted by the computer to save time and cost.

At present, the most widely used predictive methods are physical and chemical approaches, which mainly include topological polar surface area, hydrogen bond donors and acceptors, acidic and basic atomic number, ionization potential, silico methods and so on^14–19^.

**Figure 1:**
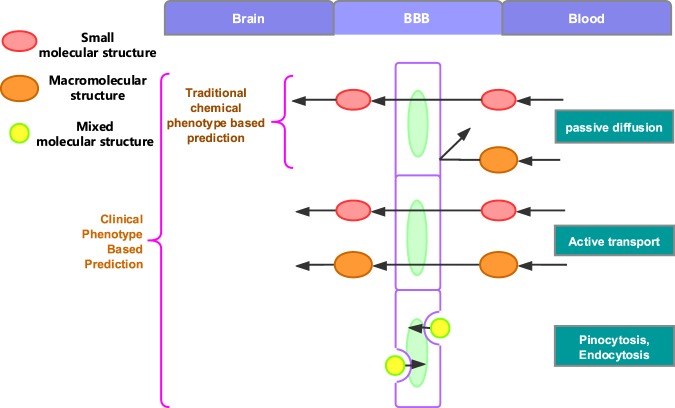
Mechanisms of drugs passing BBB and the applicable scope of prediction methods^29^. The right part presents the blood vessel, which shows the mechanisms for drug passing BBB, and the left part is the brain, which shows the scope of clinical drug phenotype based and chemical feature based BBB permeability prediction methods^29^.

Besides the physical and chemical methods, there are various supervised learning approaches, such as Support Vector Machine (SVM)^20–23^, Decision Tree (DT)^24^ and K-Nearest Neighbor (KNN)^25^ proposed for BBB drug prediction. In 2018, Wang *et al*. proposed a Silico prediction method which combines with Machine learning and resampling methods that can avoid imbalanced dataset and its accuracy of prediction reached 0.966^26^. All the methods mentioned above adopted physical or chemical features to train prediction models. In general, these methods only can be applied in small-molecule compounds that penetrate the BBB with passive diffusion. However, there are many molecules, such as glucose^27,28^, pass through BBB with more complex mechanism than passive diffusion which cannot be predicted (Fig. 1, right part). Therefore, to solve this problem, Gao *et al*. proposed a drug prediction method which based on drug side effects and drug indications^29^. This method basically solves the problem of drug entered brain with multi-mechanism and presents a new research direction of drug development for researchers (Fig. 1, left part). However, Gao only adopted multi-core SVM method and without comparing the experiment results with other methods. What’s more, the accuracy of multi-core SVM method only reached 0.76, AUC was 0.739 and F1 score was 0.76, which need to be improved urgently. For thousands of possible drugs, every 1% increase in accuracy can save a lot of drug clinical testing time. The 0.76-accuracy of existing SVM-based methods is far away to satisfy the realistic requirement.

This paper proposes a Deep Learning method in predicting the drug permeability of BBB which is based on clinical features. At present, Deep Learning method is widely used in the fields of image, sound, and text recognition, which have been achieved a majority of remarkable results. In recent years, some researchers have proposed many Deep Learning methods in the field of drug prediction and achieved excellent results^30–32^. However, the application of Deep Learning methods is still rare for the prediction of BBB permeability of CNS drugs. Therefore, our paper also tries to verify the Deep Learning method whether is effective in predicting the drug’s BBB penetration based on clinical features. Compared with the existing methods, our method has the following advantages: (i) The average prediction accuracy of experiments with three datasets already achieved 0.97, the average AUC is 0.98, F1 score is 0.91. It significantly performed better than the multi-core SVM method, Decision Tree and the KNN method, which can help researchers save experiment time and discover new drugs. (ii) The accuracy, AUC and F1 scores of SVM methods with different datasets are fluctuated greatly, but the accuracy of the Deep Learning method, which proposed in this paper, is very stable and adaptable. (iii) The Deep Learning method can be applied in both simple diffusions of small molecule compounds and other compounds that diffuse through complex pathways. In summary, this paper proposes a Deep Learning method in drug prediction of BBB permeability which is based on the clinical features and our results are better than the previous researches’ results like multi-core SVM methods. In the future, we will experiment with more types of drug data and hope our method can be applied in different disease.

The remaining sections organized Section III introduces the datasets and how we established them. Section IV is talking about the Deep Learning methods, which is design for predicting BBB permeability. Section V is the performance analyses which compared the Deep Learning method with multi-core SVM, KNN and Decision Tree (DT) on the three datasets. In section VI, it is the discussion of the advantages of the Deep Learning method proposed in this paper. Section VII concludes and describes future work.

## Results

The experiments compare the Deep Learning method with Sigmoid-Support Vector Machine (Sigmoid-SVM), POLY-Support Vector Machine (POLY-SVM), Radial Basis Function-Support Vector Machine (RBF-SVM), K-Nearest Neighbor (KNN) and Decision Tree (DT). We tested the three datasets independently. Each test randomly assigned 1000 samples into mutually exclusive training sets (70%) and validation sets (30%). We also adopt 5-fold cross validation of the training datasets and validation datasets.

We adopt several evaluation methods to ensure the precision of the results. First, we calculate the accuracy on the training and validation datasets to evaluate the learning methods. However, the accuracy is not always valid for evaluating the learning performance in different situations, especially when the true and false samples of the dataset have large difference. Then we calculate the F1 score which is an indicator used in statistics to measure the accuracy of binary classification models, and we also consider the models’ accuracy and the recall rate. Finally, in order to judge the performance of the learning models intuitively, we draw the ROC curve (Receiver Operating Characteristic curve) and calculate the AUC of the ROC curve (Area under the Curve of ROC). We also calculated all the indicators for the training and prediction datasets. Because the results analysis only requires the results of the predicted dataset, we did not list the results of the training dataset in the manuscript. The detailed results of dataset 1–3 are shown in the Supplementary Table S1

### Predictive performance of different methods with Dataset 1 and Dataset 2

**Figure 2:**
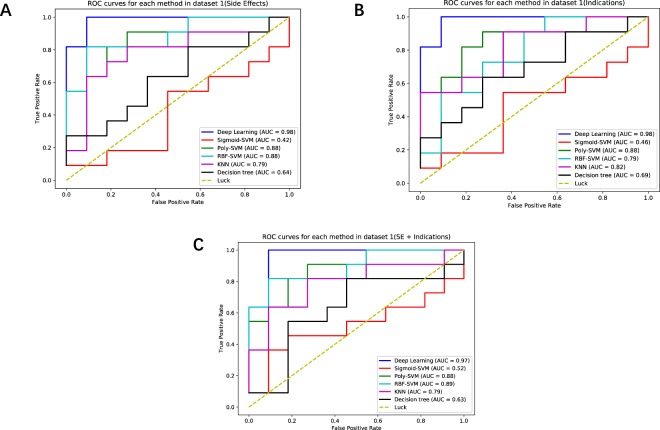
(**A**) Drug-side-effects ROC curves with different methods in the validation part of Dataset 1. (**B**) Indication ROC curves with different methods in the validation part of Dataset 1. (**C**) Drug-side-effects (SE)+ indications ROC curves with different methods in the validation part of Dataset 1.

In this section, first of all, we established Datasets 1 and 2 and validated the performance of different learning methods with them, which based on drug’s side effects, drug’s indications and drug’s side effects (SE) + indications. Then, we collected and analyzed the results of each individual test. Table 1 and Fig. 2 are the experiment outputs of Dataset 1 with different methods. According to Table 1, besides the Deep Learning method, the RBF-SVM method achieved the best results and its accuracy is 0.84, the AUC is 0.84 and the F1 score is 0.73. However, the performance of Deep Learning method is the best, the AUC increases by 13.9%, the accuracy increases by 12% and the F1 score increases by 17.4%. Therefore, the results show that Deep Learning method has better performance than the other methods on the experiments with Dataset 1.

**Table 1:** Predictive performance comparisons with different learning methods in Dataset 1.

Prediction performance in dataset 1	**Method**	**Sigmoid-SVM**			**Poly-SVM**			**RBF-SVM**		
		**Side Effects(SE)**	**Indications**	**SE + Indications**	**Side Effects(SE)**	**Indications**	**SE + Indications**	**Side Effects(SE)**	**Indications**	**SE + Indications**
		**Prediction**	**Prediction**	**Prediction**	**Prediction**	**Prediction**	**Prediction**	**Prediction**	**Prediction**	**Prediction**
	AUC	0.42 ± 0.08	0.79 ± 0.12	0.5252 ± 0.13	0.792 ± 0.12	0.81 ± 0.18	0.84 ± 0.09	0.88 ± 0.57	0.798 ± 0.21	0.84 ± 0.11
	Accuracy	0.495 ± 0.15	0.51 ± 0.11	0.52 ± 0.17	0.72 ± 0.1	0.63 ± 0.14	0.73 ± 0.08	0.89 ± 0.46	0.77 ± 0.11	0.74 ± 0.11
	F1	0.481 ± 0.17	0.5514 ± 0.12	0.607 ± 0.08	0.584 ± 0.14	0.37 ± 0.21	0.58 ± 0.11	0.76 ± 0.31	0.641 ± 0.15	0.73 ± 0.11
	**Method**	**KNN**			**Decision Tree (DT)**			**Deep Learning**		
		**Side Effects(SE)**	**Indications**	**SE + Indications**	**Side Effects(SE)**	**Indications**	**SE + Indications**	**Side Effects(SE)**	**Indications**	**SE + Indications**
		**Prediction**	**Prediction**	**Prediction**	**Prediction**	**Prediction**	**Prediction**	**Prediction**	**Prediction**	**Prediction**
	AUC	0.795 ± 0.11	0.82 ± 0.08	0.791 ± 0.13	0.64 ± 0.15	0.69 ± 0.16	0.633 ± 0.09	**0.98 ± 0.02**	**0.98 ± 0.01**	**0.979 ± 0.02**
	Accuracy	0.806 ± 0.08	0.71 ± 0.13	0.74 ± 0.11	0.574 ± 0.12	0.69 ± 0.14	0.58 ± 0.12	**0.96 ± 0.02**	**0.965 ± 0.03**	**0.96 ± 0.02**
	F1	0.712 ± 0.07	0.68 ± 0.09	0.717 ± 0.09	0.567 ± 0.12	0.661 ± 0.09	0.568 ± 0.12	**0.902 ± 0.06**	**0.891 ± 0.09**	**0.904 ± 0.04**

Each test data field shows average ± std of 1000 random splits of training and test data.

**Figure 3:**
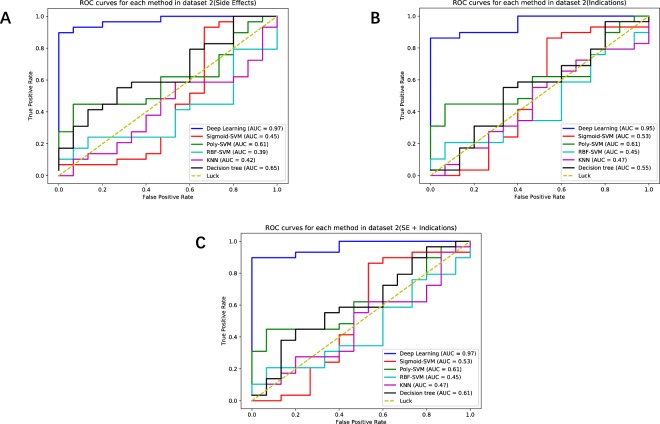
(**A**) Drug-side-effects ROC curves with different methods in the validation part of Dataset 2. (**B**) Indication ROC curves with different methods in the validation part of Dataset 2. (**C**) Drug-side-effects (SE)+ indications ROC curves with different methods in the validation part of Dataset 2.

In order to further verify the performance of the learning models, we do the experiments with Dataset 2, which has a lager sample number. The predictive performance of Dataset 2 is shown in Table 2. The drug-side-effects’, indications’ and drug-side-effects + indications’ ROC curves of Dataset 2 are shown in Fig. 3. The experimental results show that the lager the sample number of a dataset, the larger difference between the results, then the advantage of the Deep Learning method is more clear. Compared Deep Learning method with the POLY-SVM method which is performing best among the other method, the AUC increases by 31%, the accuracy increases by 44.8%, and the F1 score increases by 44.1%. More experimental details of Datasets 1 and 2 are shown in the Supplementary Tables S2 and S3.

**Table 2:** Predictive performance comparisons with different learning methods in Dataset 2.

Prediction performance in dataset 2	**Method**	**Sigmoid-SVM**			**Poly-SVM**			**RBF-SVM**		
		**Side Effects(SE)**	**Indications**	**SE + Indications**	**Side Effects(SE)**	**Indications**	**SE + Indications**	**Side Effects(SE)**	**Indications**	**SE + Indications**
		**Prediction**	**Prediction**	**Prediction**	**Prediction**	**Prediction**	**Prediction**	**Prediction**	**Prediction**	**Prediction**
	AUC	0.452 ± 0.08	0.53 ± 0.09	0.535 ± 0.13	0.6111 ± 0.08	0.61 ± 0.07	0.66 ± 0.09	0.398 ± 0.13	0.45 ± 0.09	0.45 ± 0.08
	Accuracy	0.51 ± 0.11	0.691 ± 0.13	0.52 ± 0.11	0.602 ± 0.13	0.591 ± 0.11	0.59 ± 0.24	0.41 ± 0.12	0.432 ± 0.13	0.45 ± 0.11
	F1	0.474 ± 0.13	0.51 ± 0.1	0.5221 ± 0.08	0.5201 ± 0.14	0.51 ± 0.12	0.47 ± 0.23	0.531 ± 0.14	0.512 ± 0.08	0.41 ± 0.13
	**Method**	**KNN**			**Decision Tree (DT)**			**Deep Learning**		
		**Side Effects(SE)**	**Indications**	**SE + Indications**	**Side Effects(SE)**	**Indications**	**SE + Indications**	**Side Effects(SE)**	**Indications**	**SE + Indications**
		**Prediction**	**Prediction**	**Prediction**	**Prediction**	**Prediction**	**Prediction**	**Prediction**	**Prediction**	**Prediction**
	AUC	0.421 ± 0.15	0.47 ± 0.18	0.472 ± 0.15	0.56 ± 0.15	0.51 ± 0.12	0.51 ± 0.12	**0.97 ± 0.02**	**0.9523 ± 0.03**	**0.971 ± 0.02**
	Accuracy	0.415 ± 0.16	0.445 ± 0.14	0.51 ± 0.15	0.57 ± 0.11	0.52 ± 0.15	0.52 ± 0.15	**0.9621 ± 0.02**	**0.9235 ± 0.06**	**0.968 ± 0.03**
	F1	0.541 ± 0.09	0.535 ± 0.17	0.53 ± 0.21	0.54 ± 0.15	0.56 ± 0.16	0.45 ± 0.25	**0.9008 ± 0.06**	**0.889 ± 0.08**	**0.911 ± 0.05**

Each test data field shows average ± std of 1000 random splits of training and test data.

### Deep Learning method achieved higher performance in the Independent Dataset

**Figure 4:**
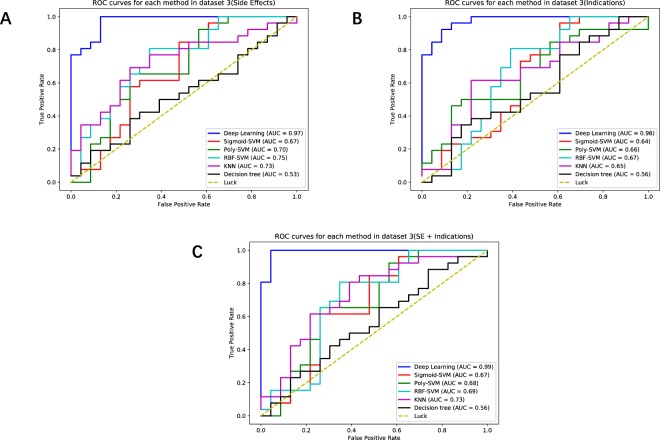
(**A**) Drug-side-effects ROC curves with different methods in the validation part of Independent Dataset (Dataset 3). (**B**) Indication ROC curves with different methods in the validation part of Independent Dataset (Dataset 3). (**C**) Drug-side-effects (SE)+ indications ROC curves with different methods in the validation part of Independent Dataset (Dataset 3).

The third experiment is using the Independent Dataset (Dataset 3), because its result can produce more accurate and more objective performance assessment. The results of different methods on the Independent Dataset are shown in Table 3. The ROC curves of Drug-side-effects, Indication, and Drug-side-effects (SE) + indications on the Independent Dataset are shown in Fig. 4 respectively. According to Table 3 and Fig. 4, we knew that the Deep Learning method still has the best performance. Compared Deep Learning method with the best performing KNN method among the other methods, the AUC increases by 25.6%, the accuracy increases by 24%, and the F1 increases 22.6%. More experimental details of the Independent Dataset (Dataset 3) are shown in the Supplementary Table S4.

**Table 3:** Predictive performance comparisons with different learning methods in Independent Dataset (Dataset 3).

Prediction performance in dataset 3	**Method**	**Sigmoid-SVM**			**Poly-SVM**			**RBF-SVM**		
		**Side Effects(SE)**	**Indications**	**SE + Indications**	**Side Effects(SE)**	**Indications**	**SE + Indications**	**Side Effects(SE)**	**Indications**	**SE + Indications**
		**Prediction**	**Prediction**	**Prediction**	**Prediction**	**Prediction**	**Prediction**	**Prediction**	**Prediction**	**Prediction**
	AUC	0.675 ± 0.15	0.72 ± 0.18	0.672 ± 0.16	0.7058 ± 0.19	0.71 ± 0.15	0.685 ± 0.12	0.71 ± 0.12	0.71 ± 0.16	0.69 ± 0.15
	Accuracy	0.51 ± 0.05	0.64 ± 0.18	0.64 ± 0.02	0.671 ± 0.14	0.53 ± 0.13	0.54 ± 0.11	0.735 ± 0.18	0.63 ± 0.12	0.69 ± 0.11
	F1	0.42 ± 0.06	0.542 ± 0.21	0.584 ± 0.09	0.521 ± 0.16	0.51 ± 0.2	0.41 ± 0.06	0.601 ± 0.21	0.62 ± 0.15	0.561 ± 0.16
	**Method**	**KNN**			**Decision Tree (DT)**			**Deep Learning**		
		**Side Effects(SE)**	**Indications**	**SE + Indications**	**Side Effects(SE)**	**Indications**	**SE + Indications**	**Side Effects(SE)**	**Indications**	**SE + Indications**
		**Prediction**	**Prediction**	**Prediction**	**Prediction**	**Prediction**	**Prediction**	**Prediction**	**Prediction**	**Prediction**
	AUC	0.73 ± 0.03	0.71 ± 0.11	0.734 ± 0.16	0.568 ± 0.16	0.67 ± 0.18	0.568 ± 0.12	**0.978 ± 0.02**	**0.98 ± 0.02**	**0.99 ± 0.01**
	Accuracy	0.7 ± 0.11	0.72 ± 0.15	0.74 ± 0.19	0.584 ± 0.2	0.66 ± 0.18	0.59 ± 0.18	**0.978 ± 0.02**	**0.964 ± 0.03**	**0.98 ± 0.02**
	F1	0.73 ± 0.07	0.71 ± 0.11	0.692 ± 0.11	0.516 ± 0.13	0.64 ± 0.16	0.519 ± 0.15	**0.91 ± 0.09**	**0.896 ± 0.1**	**0.918 ± 0.08**

Each test data field shows average ± std of 1000 random splits of training and test data.

### Inter dataset validation

To verify the versatility of the Deep Learning method, we also performed inter dataset validation. We used Dataset 2 as the training dataset and Dataset 1 as the validation dataset. The inter dataset validation results are shown in Table 4.

**Table 4:** Predictive performance comparisons with different learning methods in Inter dataset validation.

Side Effects (SE)		Indications		SE + Indications	
Training	Prediction	Training	Prediction	Training	Prediction
**Deep Learning**					
0.975 ± 0.02	0.940 ± 0.04	0.987 ± 0.01	0.980 ± 0.02	0.971 ± 0.02	0.930 ± 0.06
0.940 ± 0.05	0.901 ± 0.07	0.982 ± 0.01	0.971 ± 0.25	0.965 ± 0.03	0.910 ± 0.08
0.930 ± 0.04	0.890 ± 0.09	0.941 ± 0.09	0.920 ± 0.06	0.931 ± 0.06	0.900 ± 0.09

Each test data field shows average ± std of 1000 random splits of training and test data.

The results show that the Deep Learning method proposed in this paper achieves the ideal effect. The optimal accuracy is 0.97, the AUC is 0.98, and the F1 score is 0.92. It proves that the Deep Learning method has the best versatility among different datasets.

There is a brief summary of the experiments that the prediction accuracy of the Deep Learning method is very stable and always between 0.96 to 0.98. In addition, the best-processing method which besides the Deep Learning method is different in each dataset. At the same time, the fluctuation of accuracy is obvious which is influenced by the difference between the number of samples and the datasets and the number of positive and negative samples. What’s more, the AUC and F1 score of the Deep Learning method also remain at a relatively high level. We also performed inter dataset validation to demonstrate the versatility of the Deep Learning method. Therefore, under the conditions described in this paper, we think the performance of the Deep Learning method is better than other existing methods.

## Discussion

Research on neurological diseases has a long history. These kinds of researches can cure neurological diseases. At present, most researchers are still using various data mining algorithms based on different chemical characteristics to predict drugs’ BBB permeability^17,18^. To further improve the performance of drug prediction models, researchers are still experimenting with many new physical and chemical features such as 2D molecular descriptors and molecular fingerprints, and machine learning methods like Gaussian process, Synthetic Minority Oversampling Technique (SMOTE) and SMOTE + edited nearest neighbor^19,33–36^. In fact, to improve the predictive methods, scientists have tried more than 1,000 chemical descriptors, many of which rely on esoteric quantum chemical calculations, and it is difficult to obtain accurate data using existing techniques^37^. In addition to the reason of computational complexity, there are some situations that chemical features are not available, such as some drugs/biologic with no precisely defined structures and most of the nutrients, nutrients analogs and certain physiologically important macro-molecules which pass through BBB must with more complex biological active mechanisms^27,28,38^. According to the cases mentioned above, if a model is trained with passive diffusion of BBB agents, the accuracy of BBB penetration prediction will be low. On the other hand, scientists can neither predict the mechanism by which a drug penetrates the BBB, nor predict the applicability of the model without the support of elaborate *in vivo* experiments. In order to solve this problem, the researchers have also made many attempts, such as: trying to establish an *in vitro* model. This method will clarify the mechanism of BBB development and help researchers predict the BBB permeability of drugs^39,40^. However, these methods still cannot completely solve the problem that small molecule drugs cannot be predicted. In this case, the researchers considered using drug side effects and drug indication information to predict BBB penetrate which the advantage is that most drugs have undergone an extensive clinical application and accumulated a wealth of information. These kinds of methods can greatly broaden the prediction range of CNS drugs.

For a long time, researchers often overlooked the relation between the clinical phenotype and efficacy of CNS drugs. In order to cross this barrier, Gao *et al*. have proved that data mining methods can effectively connect these two features^29^. However, there still has a problem of prediction with data mining methods which is the accuracy relatively low which means that clinical researchers still need to spend more time and effort to verify the effectiveness of the drug.

We think that due to the difference of features based on physics and chemistry, the relation between drug side effects and adaptability is more abstract and deeper. That means traditional machine learning methods might not find the relation between data and results very efficiently, and that is the reason why the classification result is not ideal. However, basically, the characteristic of Deep Learning method is suitable for handling the data with abstract relation. To solve the problem of the small number of drugs clinical data, we try several Deep Learning Network with different depth. The results prove that these kinds of datasets are not suitable for very deep network and it requires us to build a moderate-size Deep Learning model. Therefore, the purpose of our research is trying to find out a novel classification method that can more effectively predict the drug BBB permeability based on the clinical phenotype. The experiment result validates our thought that we can get an effective relation between clinical performance and efficacy of drugs with an appropriate size and depth Deep Learning model. Because these relations are on a deep level, the results of general machine learning models are not ideal which can have better performance with Deep Learning model. The performance of Deep Learning method proposed in this paper has been proved by the experiment results that we can greatly improve the final classification results. We think the method proposed in this paper is very helpful for CNS drug calculation and saving time and cost of clinical trials.

Despite the Deep Learning method proposed in this paper has lots of advantages, it is worth noting that this method still cannot predict how the drug penetrates BBB. This is of great significance to biology. Because in this case, we cannot distinguish between the side effects and secondary effects caused by the penetration of the compound into the BBB. Therefore, in the future, we consider combining drug clinical phenotypic effects and drug chemical structure characteristics, determining the general route of drug penetration into BBB. For example, if a drug appears to be permeable in a clinical phenotype-based model and not permeable in a physical and chemical-based model, the drug may enter the body indirectly through other means.

## Conclusion

This paper proposes the Deep Learning method to predict the permeability of Blood-Brain-Barrier based on clinical phenotype. There are three datasets with independent testing and the experimental results show that the Deep Learning method performs better than multi-core SVMs, KNNs and Decision Trees. What’s more, the prediction accuracy of CNS drugs with our Deep Learning method increases more than 15%. The Deep Learning method proposed in this paper adopted the clinical phenotypic approach, which means that our method has wider applicable scope and can reduce the workload of many clinical trials of drugs.

## Materials and Methods

### Datasets of clinical drug phenotypes

According to the existing literature, this paper collected the drug names and SIDER datasets which have been proved that have BBB permeability true or false in the clinic.

The SIDER (http://sideeffects.embl.de/) dataset is a public dataset which contains a large number of drug side effects and drug indications^41^. We extracted the characteristics of the drug from this dataset. There is no existing complete BBB-permeable dataset on the Internet currently, so we refer to the literature which published in 2016^29^ that collected experimental datasets from other six academic papers^20,37,42–45^. Based on this drug dataset, we classify the drugs into two categories, one is BBB permeability true and the other one is BBB permeability false.

The clinical drug phenotypes (side effects and indications) in the SIDER database were formatted according to the Medical Dictionary for Regulatory Activities (MedDRA, http://www.meddra.org/). MedDRA divides the clinical phenotype into 5 levels: Lowest Level Term (LLT), Preferred Term (PT), High-Level Term (HLT), High- Level Group Terms (HLGT) and System Organ Classes (SOC). PT is a special descriptor and it includes the information about symptoms, therapeutic adaptability diagnosis and so on. According to the High-Level Group Terms for neurological diseases (HLGT), we selected 43 terms as clinical phenotypic characteristics of drugs and the details were listed in Supplementary Table S5. Each HLGT also contained specific side effects and indications (PT). Then, took each drug’s number of matching times under each specific HLGT group as training features^29^. More details are listed in Supplementary Table S6.

In a brief summary, we had established three datasets. The first dataset was referring to Doniger *et al*. paper which published in^20^ and this dataset had 91 samples in total, of which 38 samples were BBB permeability true and 53 samples were BBB permeability false. The second dataset was referring to the papers published from^29,37,42–44,46^ and this dataset had 210 samples in total, of which 136 samples were BBB permeability true and 74 samples were BBB permeability false. However, there was an imbalance in the sample distribution of Dataset 1 and Dataset 2. To solve the lopsidedness of the sample number of these datasets, we established the third Independent Dataset. The third dataset had 161 samples totally, of which 76 samples were BBB permeability true and 85 samples were BBB permeability false. The basic information of these datasets was shown in Table 5. The details of these datasets were given in Supplementary Tables S7–S9. The drug Side Effects and Indication based on SIDER dataset were listed in Supplementary Tables S10 and S11.

**Table 5:** The number of samples, BBB permeability true or false and data sources of the three datasets.

	Sample number	BBB permeability (true)	BBB permeability (false)	Data sources
Dataset 1	91	38	53	SIDER;^20^
Dataset 2	210	136	74	SIDER;^37,42–44,46^
Independent Dataset	161	76	85	SIDER;^29,20,37,42–44^

### System model of Deep Learning method

**Figure 5:**
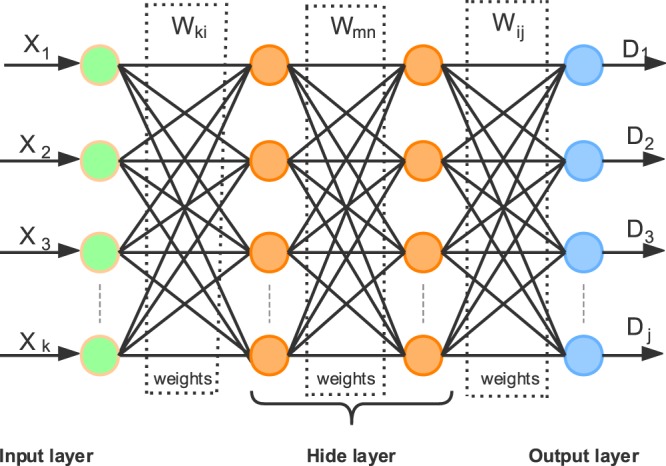
The four-layer Deep Learning model constructed in this paper, $x$ represents the data of each input node, $D$ Srepresents the data of each output node. ${W}_{ki}\,\,$is the weight between the input layer and the hidden layer, ${w}_{mn}$ is the weight between the first hidden layer and second hidden layer and ${w}_{ij}$ is the weight between the hidden layer and the output layer.

Deep Learning allows computational models that are composed of multiple processing layers to learn representations of data with multiple levels of abstraction. These methods have already dramatically improved the state-of-the-art speech recognition, visual object recognition, object detection and many other domains such as drug discovery and genomics. Deep Learning is a model that can discover the more complicated structure of datasets by using the back-propagation algorithm. According to the discovered structure, the mode can change the internal parameters. The internal parameter of each layer is the result of the previous layer^47^. For different complex datasets, the number of layers required for Deep Learning is varied. We think that although the relation between clinical side effects and adaptability of drugs may be not so strong, there may have deeper relation between clinical expressiveness and final efficacy. That means clinical expressiveness will affect final efficacy. This relation is suitable for the main idea of Deep Learning which is trying to discover the deeper relation between the data through Multi-layer Network and back Propagation algorithm. Therefore, we try to establish a Deep Learning model to verify our thought.Based on the number of samples and dimensions of the drug datasets processed in this paper, we propose the four-layer Deep Learning model to deal with these datasets. The Deep Learning model which proposed in this paper is shown in Fig. 5.

#### Hidden layer selection

The number of nodes in the input layer and the output layer of the Deep Learning network. Here we calculated the number of nodes in the hidden layer using the following equation:

1 $$h=\sqrt{m+n}+\alpha $$

Where *h* is the number of hidden layer nodes, *m* is the number of input layer nodes, *n* is the number of output layer nodes, α is an adjustment constant between 1 to 10, and generally, ${\rm{\alpha }}=1$.

#### Forward pass subprocess

Setting the weight between node $i$ and node $j$ is ${w}_{ij}$, the threshold of the node $j$ is $\,{b}_{j}$, and the output value of each node is ${x}_{j}$. The output value of each node in the current layer is changed with the output value of all nodes in the previous layer. The weights and the thresholds of the nodes are implemented by an active function. The equations are as follows:

2 $${s}_{j}=\sum _{i=0}^{m-1}{w}_{ij+{b}_{j}}$$

3 $${x}_{j}=f({s}_{j})$$

where $f$ is the active function represented by the sigmoid function, and its equation as following:

4 $$f({\rm{x}})=\frac{A}{1+{e}^{-\frac{\alpha }{\beta }}}$$

The computation procedure is from top to bottom and then from left to right, and it needs to be observed strictly to finish the entire forward process.

#### Reverse transfer subprocess

After finishing the forward pass process, we need to construct the reverse transfer process. The most important thing in the reverse transfer process is the adjustment of the weights and thresholds between each adjacent layer. The specific adjustment steps are as follows:

Step 1. Assume that all results of the output layer are ${d}_{j}$ and the equation of error function is as follows:

5 $${\rm{E}}(w,b)=\frac{1}{2}\sum _{j=0}^{n-1}{({d}_{j}-{y}_{i})}^{2}$$

Step 2. According to the gradient descent method, the weights and thresholds of the functions are modified in several times in order to minimize the error function. The gradient of $E(w,b)$ is divided by the correction of the weight vector at the current position. For the output node j:

6 $${\rm{\Delta }}w(i,j)=-\,{\rm{\eta }}\frac{\partial E(w,b)}{\partial w(i,j)}$$

Step 3. In order to calculate the weights and thresholds between the hidden layer and the output layer, we derive the active function which represents by equation (4), then through equations (7) and (8) for ${w}_{ij}$, finally ${\delta }_{ij}$ and ${b}_{j}$ are calculated by the equations (9) and (10):

7 $$\begin{array}{c}{f}^{\text{'}}(x)=\frac{A{e}^{-\frac{\alpha }{\beta }}}{B{(1+{e}^{-\frac{\alpha }{\beta }})}^{2}}\\ =\,\frac{f(x)[A-f(x)]}{AB}\end{array}$$

8 $$\begin{array}{c}\frac{\partial E(w,b)}{\partial {w}_{ij}}=\frac{1}{\partial {w}_{ij}}\times \frac{1}{2}\sum _{j=0}^{n-1}{({d}_{j}-{y}_{j})}^{2}\\ \,\,\,\,=({d}_{j}-{y}_{j})\times {f}^{\text{'}}({S}_{j})\times \frac{\partial {S}_{j}}{\partial {w}_{ij}}\\ \,\,\,\,=({d}_{j}-{y}_{j})\times \frac{f({S}_{j})[A-f({S}_{j})]}{AB}\times \frac{\partial {S}_{j}}{\partial {w}_{ij}}\\ \,\,\,\,=({d}_{j}-{y}_{j})\times \frac{f({S}_{j})[A-f({S}_{j})]}{AB}\times {x}_{i}\\ \,\,\,\,={\delta }_{ij}\times {x}_{i}\end{array}$$

9 $${\delta }_{ij}=({d}_{j}-{y}_{i})\times \frac{f({S}_{j})[A-f({S}_{j})]}{AB}$$

10 $$\frac{\partial E(w,b)}{\partial {b}_{j}}={{\rm{\delta }}}_{ij}$$

Step 4. Calculate the thresholds between two hidden layers and between the input and hidden layers. In equations (11) and (12), we suppose that ${w}_{mn}$ is the weight between the node m belongs to the first hidden layer and the node *n* belongs to the second hidden layer. The ${w}_{ki}$ is the weight between the node $K$ belongs to the input layer and the node $i$ belongs to the hidden layer. The thresholds ${\delta }_{ki}$ and ${\delta }_{mn}$ are calculated by the equations (13) and (14):

11 $$\frac{\partial E(w,b)}{\partial {w}_{ki}}=\frac{1}{\partial {w}_{ki}}\times \frac{1}{2}\sum _{j=0}^{n-1}{({d}_{n}-{y}_{n})}^{2}={\delta }_{mn}\times {x}_{m}$$

12 $$\frac{\partial E(w,b)}{\partial {w}_{ki}}=\frac{1}{\partial {w}_{ki}}\times \frac{1}{2}\sum _{j=0}^{n-1}{({d}_{i}-{y}_{i})}^{2}={\delta }_{ki}\times {x}_{k}$$

13 $${\delta }_{ki}=\sum _{j=0}^{n-1}{\delta }_{ki}\times {w}_{ki}\times \frac{f({S}_{k})[A-f({S}_{k})]}{AB}$$

14 $${\delta }_{mn}=\sum _{j=0}^{n-1}{\delta }_{mn}\times {w}_{mn}\times \frac{f({S}_{m})[A-f({S}_{m})]}{AB}$$

Step 5. According to the gradient descent method and the formulas, which mentioned above, equations (15) and (16) are used to adjust the weights and thresholds between the hidden layer and the output layer. The equations (17) and (18) are used to adjust the weights and thresholds between two hidden layers. The equations (19) and (20) are used to adjust the weights and thresholds between the input layer and the hidden layer:

15 $${w}_{ij}={w}_{ij}-\eta \times \frac{\partial E(w,b)}{\partial {w}_{ij}}={w}_{ij}-{\eta }_{1}\times {\delta }_{ij}\times {x}_{i}$$

16 $${b}_{j}={b}_{j}-{\eta }_{2}\times {\delta }_{ij}$$

17 $${w}_{mn}={w}_{mn}-{\eta }_{1}\times {\delta }_{mn}\times {x}_{mn}$$

18 $${b}_{n}={b}_{n}-{\eta }_{2}\times {\delta }_{mn}$$

19 $${w}_{ki}={w}_{ki}-{\eta }_{1}\times {\delta }_{ki}\times {x}_{k}$$

20 $${b}_{i}={b}_{i}-{\eta }_{2}\times {\delta }_{ki}$$

There is the whole procedure of the reverse transfer process in the Deep Learning method which is proposed in this paper. To complete the learning process of the entire Deep Learning network, the continuous adjustments of weights and thresholds are necessary. We can set an error threshold or a maximal number of cycles as a stop criterion to break off the entire learning process.

### Related methods for evaluation

Nowadays, there are many usual methods in predicting the drug permeability of BBB, such as multi-core SVM, KNN, DT and so on. Therefore, we select several methods to compare with the Deep Learning method, which proposed in this paper in order to evaluate the performance of our method.

#### Multi-core SVM method

Multi-core SVM method is one of the most common methods in the published BBB permeability papers. For example, Gao *et al*. adopted POLY-SVM, RBF-SVM and normalized POLY-SVM methods in predicting the drug permeability of BBB^29^.

The SVM method assumes the hyperplane equation is ${w}^{T}+b=0$. Let $x$ be a vector of $N$ dimensional input space. Let $\varnothing (x)=({{\rm{\phi }}}_{1}(x),{{\rm{\phi }}}_{2}(x),\,.\,.\,.,{{\rm{\phi }}}_{M}(x))\,$denote the nonlinear transformation from the input space to the M-dimensional feature space. A superclass plane can be constructed in this feature space and the equation is^48^:

21 $$\sum _{j=1}^{M}{w}_{j}{\varnothing }_{j}(X)+b=0$$

where ${w}_{j}$ is the weight that connects the feature space to the output space, and $b$ is the offset.

If the data is not linearly separable, the kernel function will be used. The common kernel functions include Linear, Poly, RBF, Sigmoid and so on. Gao *et al*. paper proposed to use POLY-SVM, RBF-SVM and normalized POLY-SVM method in predicting the drug permeability of BBB which is based on clinical features^29^. However, in normalized POLY-SVM, the normalization only uses to preprocess the data and its influence on the results is slight. Therefore, we use another high performing method named Sigmoid-SVM method instead of normalized POLY-SVM in comparison.

#### Drug prediction with KNN method

KNN method is a kind of the classical data mining methods and it also has been used to predict drug penetration of BBB in many years.

KNN method is measuring the distance between different feature values. Its main idea is that if a sample in the feature space, most similar samples of $K$, which means the nearest neighbors in the feature space, belong to a certain category, then the sample also belongs to this category, where $K$ is usually not greater than an integer of 20^25^.

#### Drug prediction with Decision Tree (DT)

Decision Tree (DT) looks like the tree structure, which can be a binary tree or a non-binary tree. Each non-leaf node represents a feature attribute, each branch represents the output of the feature attribute in a range of values, and each leaf node stores a category^27^.

DT begins at the root node, then judge the corresponding feature in the item to be classified and selects the output branch according to its value until it reaches the leaf node. Finally, DT saved the category at the leaf node as the result of the decision^49^.

## Supplementary information

Table S1

Table S2

Table S3

Table S4

Table S5

Table S6

Table S7

Table S8

Table S9

Table S10

Table S11
